# Genome of Russian Snow-White Chicken Reveals Genetic Features Associated with Adaptations to Cold and Diseases

**DOI:** 10.3390/ijms252011066

**Published:** 2024-10-15

**Authors:** Ivan S. Yevshin, Elena I. Shagimardanova, Anna S. Ryabova, Sergey S. Pintus, Fedor A. Kolpakov, Oleg A. Gusev

**Affiliations:** 1 Biosoft.Ru, LLC., 630058 Novosibirsk, Russia; ivan@biosoft.ru (I.S.Y.);; 2 Life Improvement by Future Technologies (LIFT) Center, 121205 Moscow, Russia; 3 Center of Genomics and Bioimaging Core Facility, 121205 Moscow, Russia; 4 Sirius University of Science and Technology, 354340 Sirius, Russia; sspintus@biosoft.ru (S.S.P.);; 5 Regulatory Genomics Research Center, Institute of Fundamental Medicine and Biology, Kazan Federal University, 420008 Kazan, Russia; 6 Intractable Disease Research Center, Graduate School of Medicine, Juntendo University, Tokyo 13-8421, Japan

**Keywords:** chicken, genome, cold adaptation, disease resistance

## Abstract

Russian Snow White (RSW) chickens are characterized by high egg production, extreme resistance to low temperatures, disease resistance, and by the snow-white color of the day-old chicks. Studying the genome of this unique chicken breed will reveal its evolutionary history and help to understand the molecular genetic mechanisms underlying the unique characteristics of this breed, which will open new breeding opportunities and support future studies. We have sequenced and made a de novo assembly of the whole RSW genome using deep sequencing (250×) by the short reads. The genome consists of 40 chromosomes with a total length of 1.1 billion nucleotide pairs. Phylogenetic analysis placed the RSW near the White Leghorn, Fayoumi, and Houdan breeds. Comparison with other chicken breeds revealed a wide pool of mutations unique to the RSW. The functional annotation of these mutations showed the adaptation of genes associated with the development of the nervous system, thermoreceptors, purine receptors, and the TGF-beta pathway, probably caused by selection for low temperatures. We also found adaptation of the immune system genes, likely driven by selection for resistance to viral diseases. Integration with previous genome-wide association studies (GWAS) suggested several causal single nucleotide polymorphisms (SNPs). Specifically, we identified an RSW-specific missense mutation in the RALYL gene, presumably causing the snow-white color of the day-old chicks, and an RSW-specific missense mutation in the TLL1 gene, presumably affecting the egg weight.

## 1. Introduction

Chicken is the most widespread poultry, widely used for food production and as model organisms for studying the mechanisms of embryonic development. Over the long history of human domestication, a large number of breeds of chicken have been developed. According to the Domestic Animal Diversity Information System of the Food and Agriculture Organisation (FAO), currently, there are more than 1500 chicken breeds (https://www.fao.org/dad-is/browse-by-country-and-species/en/ accessed on 1 August 2024). In recent years, the genomes of chickens have been actively studied; currently, the genomes of 26 breeds have been sequenced [https://www.ncbi.nlm.nih.gov/datasets/genome/?taxon=9031 accessed on 1 August 2024]. Such a collection gives an opportunity to comprehensively analyze the genetic features associated with unique breed-specific features in morphology, physiology and other aspects.

The Russian White chicken breed was created in the USSR by crossing the White Leghorn with local Russian breeds. The RSW breed was derived from the Russian White, and during 1954–1970 was subjected to a high degree of inbreeding and selection for extraordinary resistance to the cold and some diseases, like viral leukemia and Marek’s disease [1,2]. Selection for cold resistance took place over 25 generations based on the principle of chicken survival in the first five days of life at low temperatures. As a result, RSW chickens have become cold-resistant and are able to survive from a day-old age at temperatures of 16 °C, and adult chickens can be kept in unheated poultry houses in the winter [1,2]. The creation of a breed by selection for the traits of interest should have been reflected in the genome, which is of great interest to researchers, since genome research can shed light on the molecular mechanisms of resistance to cold and disease [1,2,3,4,5,6].

To date, research on the genome of the Russian White and its derived population RSW has been limited to the individual genes and genome-wide SNP (single nucleotide polymorphism) chips. In this way, several genomic loci associated with unique RSW characteristics have been identified [3,4,6,7]. At the same time, the unusual tolerance of the RSW to cold and the remarkable phenotypic changes compared to other chicken breeds suggest strong changes in the genome. The de novo assembly and annotation of the RSW would provide a new, promising layer of knowledge about the link among the structure of the genes and the regulatory elements in the genome resulting in such drastic physiological changes. In this paper we sequenced, assembled, annotated, and analysed the genome of the RSW for the first time and revealed several strong unique features of the genome to be associated with the unique properties of this breed and the evolution of resistance to unfavourable environmental conditions in birds in general.

## 2. Results

### 2.1. Sequencing, Assembly, and Annotation of RSW Genome

**Figure 1:**
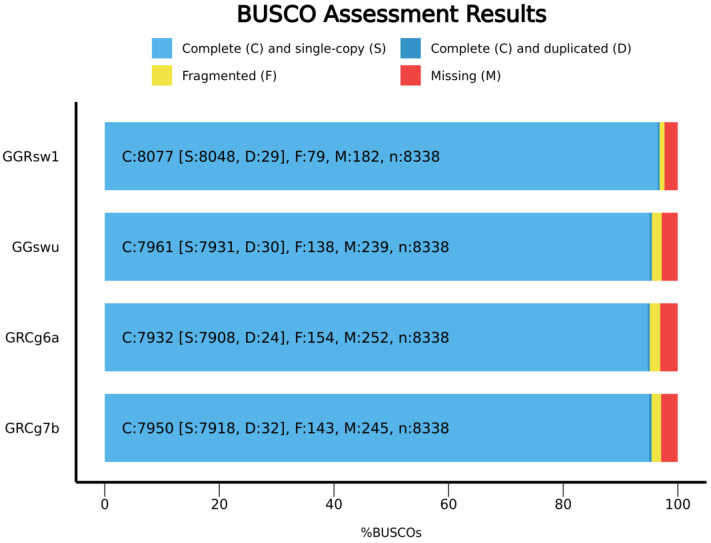
The BUSCO assessment results for the 4 genome assemblies, showing the percentages and categories of the single-copy orthologs from the aves_odb10 data set (total genes = 8338) in each genome assembly: GGRsw1—the genome assembly of the RSW in this study; GGswu—the genome assembly of the Huxu breed [9]; GRCg6a—the genome assembly of the Red Junglefowl (official reference genome from the Genome Reference Consortium); and GRCg7b—the genome assembly of the broiler (official reference genome from the Genome Reference Consortium). GGRSw1 contains a greater number of single-copy orthologs, and lower numbers of missing and fragmented genes.

The genome of one RSW chicken was sequenced using short paired reads with a high coverage. In total, 963.9 million pairs of reads were sequenced, corresponding to a 250× genome coverage. FASTQC [8] showed a high quality of sequenced reads. After the de novo assembly of the paired reads, we obtained 54,114 contigs of a total length of 1.049 Gbp. The N50 value was 194,493 bp, which is better than the 10 other illumina-based assemblies (85–143 Kbp) available in the NCBI genomes database (Table S12). The L50 value was 1508 and the overall GC percent was 42.2%. To obtain a chromosome-level assembly, we ordered the contigs according to the Huxu breed reference genome [9]. As a result, we obtained the sequences of thirty-eight autosomal and two sex chromosomes W, Z of a total length of 1095 Mbp. The mitochondrial genome, 16,785 bp in length, was assembled separately and added to the final assembly (see Section 4). The contigs that were not placed into the known chromosomes were cleared of non-bird sequences and merged into the artificial chromosome named chr_unplaced. The total length of the unplaced contigs after filtering was 27.7 Mb. The final assembly, named GGRsw1, is available for download at https://chicken.biouml.org/downloads/ChickenResearch2023/RussianSnowWhite/GGRsw1_genomic.fa.gz (accessed on 1 August 2024). The BUSCO completeness of the GGRsw1 assembly was 96.8% (Figure 1), which is higher than the completeness of the current reference genomes, GRCg6a (95.1%), GRCg7b (95.4%), and GGswu (Huxu 95.5%). We annotated 24,647 genes in the GGRsw1 assembly including 17,431 protein-coding genes, 5679 lncRNA, and 794 miRNA. All annotations are available for download in gff3 format at https://chicken.biouml.org/downloads/ChickenResearch2023/RussianSnowWhite/ggrsw1_genes.gff3.gz (accessed on 1 August 2024).

The diploid nature of the chicken genome allowed us to recover the heterozygous sites of the sequenced chickens that at least partially reflect the diversity within the RSW breed. In total, 3.4 million variants were identified, including 3.15 million SNPs and 289.5 thousand small insertions and deletions. The resulting BCF file is available for download at https://chicken.biouml.org/downloads/ChickenResearch2023/RussianSnowWhite/heterozygous_sites.bcf (accessed on 1 August 2024).

### 2.2. Comparison with Genomes of Other Breeds

At the time of the study, 33 chicken genomes belonging to 24 breeds were available in the NCBI Genomes database (Table S12). To identify the specific characteristics of RSW chickens, we performed a pairwise alignment of the genomic sequences of GGRsw1 with the 33 chicken genomes of the other breeds. We observed a high homology of RSW sequences with sequences from other breeds: the per base divergence ranged from 0.44% to 0.61%. The most similar breed was the White Leghorm with 4.4 million substitutions, and the most distant was the Thailand Gamefowl with 6 million substitutions. The GGRsw1 assembly covers almost all known chicken genomic sequences; namely, it covers 97.9% of the White Leghorn assembly GCA_024679905.1, 97.5% of the Huxu assembly GGswu, and 96.5% of GRCg7b.

**Figure 2:**
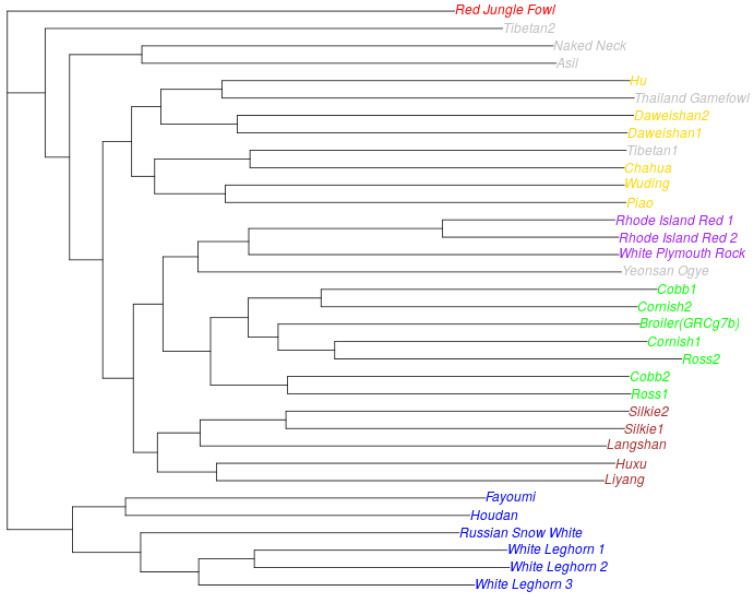
A phylogenetic tree based on the comparison of the whole genome sequences of chickens of different breeds. Red—The Red Junglefowl is a chicken from Southeast Asia, from which domestic chickens probably originate; blue—“European” breeds; purple—American breeds; green—broiler breeds; yellow—Chinese breeds from Yunnan province; Brown—Chinese breeds not of Yunnan origin; and gray—other breeds.

Phylogenetic analysis of the entire genome shows the evolutionary relationship of the RSW breed to the White Leghorn, forming together with the old French Houdan breed and the ancient Egyptian Fayoumi breed a distinct group (Figure 2).

The resulting phylogenetic tree also shows the existence of several other breed groups:

- Broiler breeds (Cornish, Cobb, Ross, GRCg7b)
- American breeds (Rhode Island Red, White Plymouth Rock) together with an ancient Korean breed (Yeonsan Ogye)
- Chinese breeds not of Yunnan origin (Silkie, Langshan, Huxu, Liyang)
- Chinese breeds from Yunnan province (Daweishan, Hu, Piao, Wuding, Chahua) together with the Thailand Gamefowl and one of the Tibetan chickens.

To characterize the specific features of the RSW genome, we identified mutations specific to this breed and not found in the other breeds that have a sequenced genome (see Section 4). In this way, 100,946 RSW-specific variants were found.

In order to characterize the functions specifically altered in the RSW, we matched the RSW-specific variants to gene annotations. Analysing the protein-coding genes, we found 1107 genes with nonsynonymous substitutions [Supplementary Table S8]. Additionally, we identified genes with a high rate of nonsynonymous mutations (Table 1) (fold > 2, *p*-value < 1 × 10^−3^) (see Section 4.1 for the method of fold change and *p*-value calculation). Of particular interest are the genes with the highest rates of nonsynonymous mutations in the RSW: *T-cell receptor alpha variable 20-like* and *INPP5D (SHIP1)*. *T-cell receptor alpha variable 20-like* is a gene encoding short protein (148 amino acids) that has five nonsynonymous mutations specific to the RSW, which is 28.5 times higher than on average. *INPP5D (SHIP1)* has three RSW-specific missense mutations in the coding part of the gene that are 35 times higher in mutation rate than the average mutation rate of this gene in other chicken breeds.

Similarly, we identified 1014 genes with a high rate of RSW-specific mutations in the ±10 kbp region of the gene bounds (Supplementary Table S9) (fold > 2, *p*-value < 1 × 10^−3^). 

To characterize the function of the genes altered in the RSW breed, we performed an enrichment analysis of the gene ontology categories [Supplemental Tables S10 and S11]. The classic “gene list” approach is not suitable in this case because it systematically favors longer genes that randomly have more mutations. So, we developed a special GO enrichment analysis described in “Materials and Methods. Section 4.1”. Among the enriched gene ontology categories, there are many categories related to the nervous system, thermoreceptors, purine receptors, TRP ion channels, TGF-beta pathway, MHC complex, and B-cell immune response. The RSW breed has altered genes in the development pathways of the nervous system. Missense mutations in the gene ontology genes of the “nervous system development” group are found 2.1 times more often in the RSW than in other chicken breeds (*p*-value = 4.4 × 10^−8^) (see Tables S1 and S10). Furthermore, an increase in the frequency of mutations occurs in the similar groups of genes “neuron development”, “neuron differentiation”, “neurogenesis”, and “generation of neurons” (see Table S10). Many TRP ion channels are specifically mutated in the RSW breed. We found seven RSW-specific mutations in the coding parts of the TRP genes: *TRPC4*, *TRPM1*, *TRPM3*, *TRPM6*, *TRPM7*, *TRPV1*, and *TRPV2*. Unexpectedly, all seven mutations turned out to be nonsynonymous. RSW chickens have specific mutations in the purinergic signaling pathway. We found an increased frequency of missense mutations in the genes in the GO category “response to purine containing compounds” (fold change 8.1, *p*-value 2 × 10^−6^). The genes with RSW-specific missense mutations include *P2RX4*, *HCN1*, *TLR7*, *P2RX7*, and *PKD2*. Our comparative analysis of chicken genomes showed that the genes involved in the TGF-beta pathway have a higher frequency of missense mutations in the RSW breed compared to other chicken breeds (Table 2 and Table 3). We found an excess frequency of RSW-specific mutations in the genes associated with the immune system (Table 4 and Table 5). In total, we found 13 GO categories related to immunity [Table 4] and 55 genes [Table 5] corresponding to these categories specifically mutated in the RSW.

#### Identification of RSW-Specific Genomic Regions

**Figure 3:**
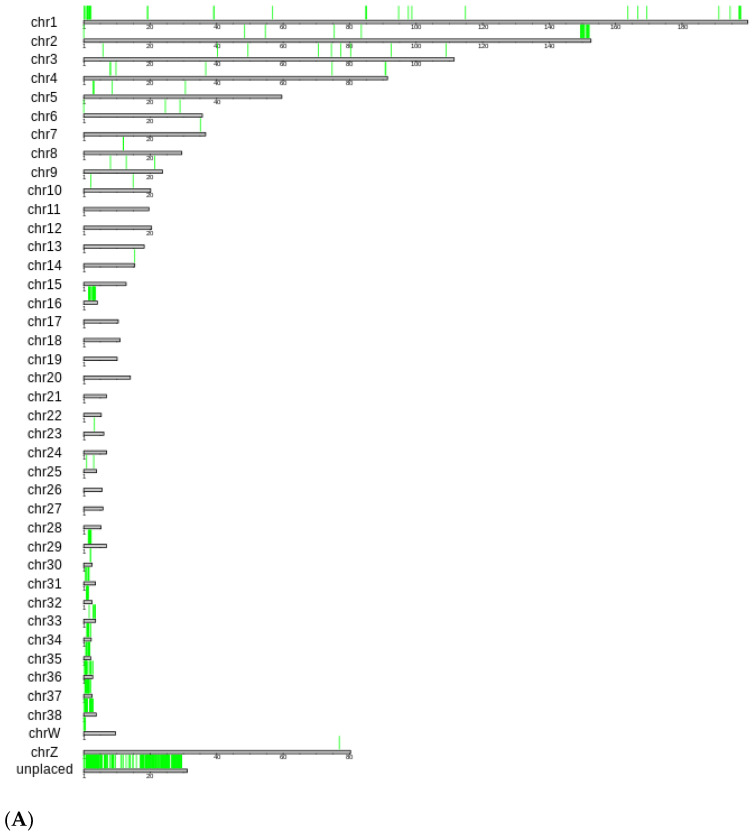

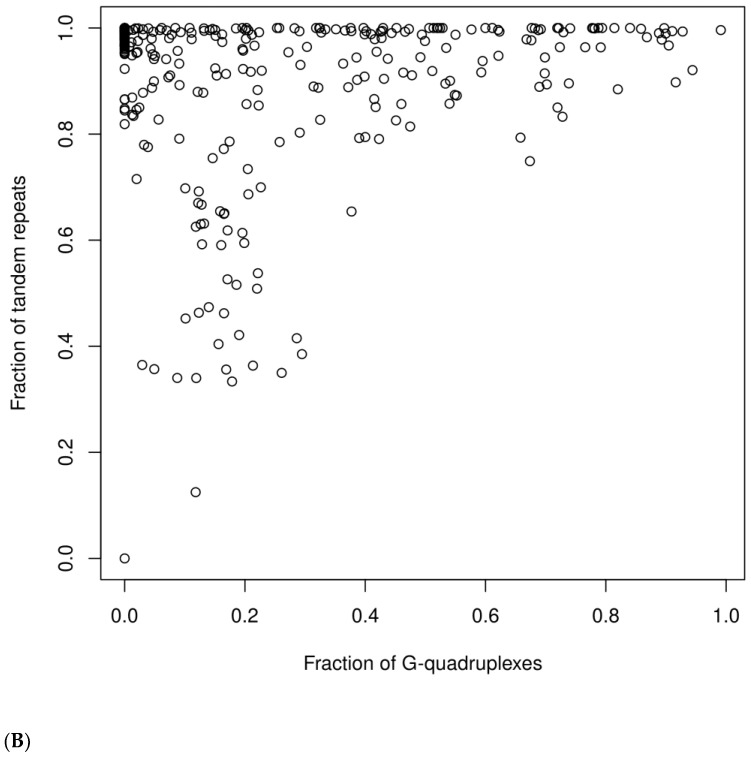
(**A**). The genomic regions unique to the Russian Snow White longer than 1000 bp. A map of all GGRsw1 chromosomes is shown, with green bars marking the genomic regions unique to the Russian White breed. (**B**). The figure shows, for each RSW-specific sequence, what proportion of that sequence is composed of G4s and tandem repeats: X axis—the fraction of G4s; Y axis—the fraction of tandem repeats.

By comparing the genome of the RSW with the genomes of other chicken breeds, we identified long regions in the GGRsw1 genome that are absent from the genomes of the other breeds. A total of 352 regions ranging in length from 1000 to 4857 nucleotide pairs were identified. These unique genomic sequences of the RSW are not evenly distributed throughout the genome [Figure 3A]. Most of them are located in the microchromosomes (chr16, chr29–chr38) and in the telomeric regions of the chromosomes chr1 and chr2. The content analysis of these sequences revealed the presence of a large number of tandem repeats (90.5% in these regions versus 0.89% in the whole genome) and G-quadruplex secondary structures (G4s) (23.2% in these regions versus 3.7% in the whole genome) [Figure 3B]. The percentage of G4s is higher in RSW-specific regions from the known chromosomes (31.2% on average) than in the regions from unplaced contigs (19.3%), but the percentage of tandem repeats is the same. Only two genomic regions contain zero or moderate numbers (<20%) of tandem repeats and G4s. The only RSW-specific region without tandem repeats and G4s is a 1441 bp sequence located in a cluster of myosin heavy chain genes on chr18 between the *MYH1C* and *MYH1E* genes [Supplemental File S1]. By aligning this sequence with the Conserved Domain Database, we identified a similarity to the motor domains of the class II myosin heavy chain 3 and class II myosin heavy chain 13 proteins, indicating the possible presence of a gene from the myosin heavy chain family. It is interesting that a similar sequence is present in the genomes of *Tetrao urogallus* (capercaillie) and *Meleagris gallopavo* (turkey) (100% coverage at 89% identity) but not in the genomes of the other chicken breeds, indicating the possible loss of this sequence in many chicken breeds, but not in the RSW. Another 1762 bp length RSW-specific genomic region contains 12% of tandem repeats and 12% of G4s, located on the chr2 upstream of the *OPLAH* gene (5-oxoprolinase, ATP-hydrolysin) (Supplemental File S2). Alignment to the Conserved Domain Database showed the existence of *OPLAH* domains in this sequence.

### 2.3. RSW-Specific Mutations in Genomic Regions Associated with Phenotypic Traits

Previously, several genomic associations with phenotypic traits were found using the SNP chip approach in the RSW breed [6]. A large locus (several megabases) on chr2 was found to be associated with the white colour of the day-old chick, and with the extraembryonic fluid production. Both of these traits are a unique feature of the RSW breed and, therefore, are probably caused by RSW-specific mutations. By genome sequencing, we found two missense RSW-specific mutations in this region in the genes *ZFHX4* and *RALYL* (Table 6). These mutations do not occur in other breeds of chicken and were therefore missed by the SNP chips.

The genomic region near the *TLL1* gene was previously associated with egg weight in the Russian White breed [6]. We found an RSW-specific chr4:23748362:C > A missense mutation of the *TLL1* gene that is a great candidate for the causative SNP for the egg weight.

### 2.4. RSW-Specific Mutations in Genomic Regions under Selection Pressure

Previously, several studies [3,4,7] investigating the genome of the Russian White chicken with SNP chips discovered genomic regions that were recently under selection pressure and discussed their connection with adaptation to cold conditions. By genotyping 20 chickens of the Amroks breed and 177 chickens of the Russian White breed using SNP chips, 12 run of homozygosity (ROH) islands specific to the Russian White were identified, presumably related to cold adaptation [3]. By sequencing the genome, we found 1615 RSW-specific mutations in these regions [Supplementary Table S2], which are good candidates for the role of causal SNPs. In a similar study [7], 31 Russian White genotypes were compared to 23 White Cornish genotypes, and seven ROH regions specific to the Russian White were detected. We found 343 RSW-specific variants in these regions [Supplementary Table S3]. Recently, Romanov et al. [4] analysed the genotypes of 156 chickens from four breeds (Russian White, Ushanka, Orloff Mille Fleur, and White Cornish) and identified 51 genomic regions under selection in the Russian White. We found 2329 RSW-specific variants in these regions (Supplementary Table S4). Furthermore, most of the genomic regions identified in the aforementioned studies did not show an increased density of RSW-specific SNPs, and few of them have a density 1.4–4.7 times higher than the genomic average (Table 7). Additionally, we identified 73 protein-coding genes that have a missense RSW-specific mutation in these regions [Supplementary Table S5] and 56 genes that have an increased rate of RSW-specific mutations in the nearby area (±10 kb from the gene boundaries) [Supplementary Table S6].

## 3. Discussion

Although the chicken genome is relatively small (1.1 Gb), its sequencing poses certain challenges. Thus, some genes remain invisible when the genomic DNA is sequenced, even though they are visible when the RNA transcripts are sequenced [10]. The reason appears to be the large number of GC-rich regions and G4s in the DNA structure, which reduces the efficiency of the PCR amplification of these regions [11,12]. To overcome this problem, the depth of the sequencing was artificially increased by integrating sequencing data from different breeds and creating a so-called pangenome [11], which is an addition to the reference genome sequence of 159 million base pairs in length. Hidden GC-rich regions of the Huxu chicken breed genome have also been successfully sequenced using Nanopore sequencing technology, which is less susceptible to this problem [9]. Increasing the sequencing depth to 250× allowed us to recover most of the RSW genome, as evidenced by the high BUSCO completeness (96.8%), greater than in the reference assemblies GRCg6a, GRCg7b, and GGswu. The high quality of the GGRsw1 assembly allows it to be used as a reference genome in future studies. However, there are 2.5% of unplaced sequences in the genome assembly, and this could be refined in future studies using long-read sequencing methods, Hi-C, and optical mapping. Furthermore, we provide a gene annotation for the GGRsw1 assembly that contains most of the known chicken genes. The gene annotation can also be improved in the future by sequencing the transcriptome under various experimental conditions and tissues. GGRsw1 is the first genomic assembly of a unique RSW breed, which opens up great opportunities for future research. We sequenced only one chicken and were able to reconstruct its heterozygous sites, which opens the door to understanding the genetic diversity within the RSW breed, but more research is necessary in this area.

Phylogenetic analysis of the RSW genome has shown its relationship with European and Mediterranean chicken breeds, apparently separated early from the other breeds in the history of domestication. The relationship of the RSW to the White Leghorn breed is consistent with the historical data on its origin. We have established a relationship with the old French Houdan breed and the ancient Egyptian Fayoumi breed for the first time. The relationship of the Russian White with another French breed, the Faverolles Salmon, was obtained earlier based on multilocus DNA fingerprinting [13].

A comparative analysis of the RSW genome with the genomes of the other chicken breeds revealed a large number of RSW-specific mutations. Such RSW-specific mutations were expected to reflect the unique characteristics of this breed, such as the cold resistance, the resistance to viral diseases, and the snow-white colour of the day-old chicks. Analysing the RSW-specific mutations, we found an excess frequency of mutations in genes from the pathways responsible for body temperature maintenance. The altered thermoregulation pathways include the nervous system, thermoreceptors, purine receptors, and the TGF-beta pathway. This can be explained by the artificial selection for cold resistance during RSW breeding. The control of body temperature is carried out by the nervous system. It is also known that the development of an organism under cold conditions triggers the mechanisms of adaptation to the cold through the modulation of neuronal differentiation [14,15]. The mechanism of thermosensitivity of the neurones is based on the action of thermosensitive TRP ion channels. When the temperature changes, the TRP ion channels can alter the entry of ions into the cell, which can lead to changes in the membrane potential [16]. We discovered a large number of RSW-specific mutations in the genes associated with neuronal differentiation and growth. Furthermore, seven TRP ion channels are specifically mutated in the RSW breed. Four of them are high temperature receptors (*TRPV1*, *TRPV2*, *TRPM3*, *TRPC4*) [17,18,19]. The remaining *TRPM1,6,7* channels have no known function in temperature sensitivity. Interestingly, we did not detect RSW-specific mutations in the coding parts of the cold-sensitive receptor genes *TRPM8* and *TRPA1*. The purinergic receptors *P2X* are cation channels activated in response to extracellular ATP, with important roles in various biological functions. We found an increased frequency of mutations in genes from the purinergic signaling pathway. The genes specifically mutated in the RSW include *P2RX4*, *HCN1*, *TLR7*, *P2RX7*, and *PKD2*, many of which are involved in body temperature maintenance. *P2RX7* knockout mice have a lower core body temperature and an increased expression of *P2RX4* mRNA in the hypothalamus [20]. *HCN1* is a hyperpolarization-activated cyclic nucleotide-gated channel that functions in the heart and central nervous system, including the hypothalamus. *HCN1* has been shown to be involved in long-term cold adaptation in mice [21]. *HCN* channels have also been linked to cold perception. That is, *HCN1* shows increased expression in oxaliplatin-induced cold hypersensitivity, and its specific inhibitor ivabradine abolishes cold hypersensitivity [22]. Also, *PKD2* (also known as *TRPP1*) was not implicated in body temperature regulation; it is a member of the transient receptor potential (TRP) family, many of which are well-known temperature sensors. We hypothesise that the genetic adaptations to cold in the RSW may be directed toward modulating the nervous system.

The transforming growth factor beta (TGF-beta) signaling pathway participates in many cellular processes, including thermoregulation and immune response [23,24]. We found that the genes involved in the TGF-beta pathway have an increased frequency of RSW-specific mutations (Table 2 and Table 3). TGF-beta is involved in the thymic selection of T cells and in the homeostasis of the naive T-cell pool. Also, TGF-beta controls the development of B cells, natural killer cells (NKs), macrophages, dendritic cells, and granulocytes [24,25]. In addition, many of the components of TGF-beta are involved in thermoregulation [23]. We hypothesise that these mutations in the genes of the TGF-beta pathway may appear due to selection for resistance to viral diseases or cold conditions.

The RSW breed has been selected for resistance to viral leukemia and Marek’s disease [1,2], which could be reflected in its genome. In fact, we found an excess frequency of RSW-specific mutations in the genes associated with the immune system (Table 4 and Table 5). Interestingly, this excess of mutation frequency is observed near the genes (±10 kb) but not in the coding regions, indicating the possible involvement of transcriptional regulation. Marek’s disease (MD) is a highly contagious viral disease caused by *Gallid alphaherpesvirus 2* (GAHV-2) that results in the rapid development of T-cell lymphomas in chickens [26]. Additionally, RSW chickens were selected for resistance to leukemia caused by the *Rous sarcoma virus* (RSV) [2]. The RSV is a retrovirus with a single-stranded RNA genome that can integrate into the genome of chickens through reverse transcriptase and integrase enzymes. Interestingly, we discovered the integration of the RSV into the RSW genome, as well as into the genomes of other breeds (Huxu, GRCg7b), which may affect the susceptibility of these breeds to this virus. Among the most mutated genes in the RSW breed are the genes related to immunity, *T-cell receptor variable 20-like*, *mitochondrial antiviral signaling protein* (*MAVS*), and *INPP5D (SHIP1)* [27], which may be caused by selection for the resistance to viral diseases. In addition to the role of purinoceptors in thermoregulation, purinoceptors are also involved in immunity. That is, the *P2RX7* receptor is crucial for controlling how antigens are presented and how T cells are activated [28]. Furthermore, many purinergic receptors, including *P2RX4* and *P2RX7*, have distinct expression patterns in MD-resistant and MD-sensitive chicken breeds during MD infection [29], indicating that the adaptation of purinergic signaling may be due to the selection for MD resistance in the RSW breed.

In addition to the RSW-specific SNP analysis, we identified long RSW-specific genomic regions, most of which are rich in tandem repeats and G4s. The high content of G4s in the RSW-specific regions may be explained by the fact that they were missed previously when they were sequenced with a low sequencing coverage. The tandem repeats are likely to have evolved through unequal crossing-over and strand slippage during DNA replication [30]. The only such genomic region without tandem repeats and G4s contains a gene of the myosin heavy chain family, which has apparently been lost in most chicken breeds except the RSW. Another RSW-specific genomic region with a low concentration of tandem repeats and G4s apparently contains an additional 5′ exon of the *OPLAH* gene, since the known chicken *OPLAH* mRNA (XM_040696025 970 bp) is significantly shorter than the similar human mRNA (XM_047421688 4289 bp). We cannot determine whether this sequence has been lost by many breeds of chicken or whether it is missing from the genome assemblies due to sequencing difficulties, as it contains a significant number of G4s.

A search for RSW-specific SNPs in the regions previously identified by GWAS in the RSW yielded two good candidate causal SNPs for the snow-white trait in the *ZFHX4* and *RALYL* genes, and for the egg weight trait in the *TLL1* gene. Furthermore, the *RALYL* gene is located in the genomic region that presumably was recently under selection pressure [3]. Interestingly, using a GWAS, a connection between the *RALYL* gene and the colour of duck feathers was recently discovered [31], supporting the idea of its role in aves colour. Therefore, the missense mutation of the *RALYL* gene (chr2: 122881444: G > T) may play a key role in the pigmentation of day-old RSW chicks, but the molecular mechanisms require further study.

## 4. Materials and Methods

The tissue samples of the RSW chickens were provided by the Russian Research Institute of Farm Animal Genetics and Breeding. The genomic DNA was isolated from the liver tissue of one chicken using a DNeasy Blood & Tissue Kit (Qiagen, Hilden, Germany). 250 ng of total DNA was used for the paired-end DNA library preparation using the NEBNextUltra II DNA Library Prep Kit (New England Biolabs, Ipswich, MA, USA) and NEB Next Single Index set (New England Biolabs, Ipswich, MA, USA) according to manufacturer instructions. Whole-genome sequencing was carried out by the NovaSeq6000 sequencing platform (Illumina, San Diego, CA, USA) with a 2 × 150 bp read length. Sequencing was performed at the Skoltech Genomics and biovisualization Core Facility.

Read quality control was conducted using FASTQC v0.11.8 [8]. Sequencing adapters were identified in 2.1% of the read pairs. The adapter sequences were:

Adapter1:

AGATCGGAAGAGCACACGTCTGAACTCCAGTCACNNNNNNATCTCGTATGCCGTCTTCTGCTTG

Adapter2:

AGATCGGAAGAGCGTCGTGTAGGGAAAGAGTGTAGATCTCGGTGGTCGCCGTATCATT.

The adapters were removed before the subsequent mapping to the genome and were kept for the de novo assembly procedure.

As part of the preliminary analysis before the de novo genome assembly, we mapped the sequenced reads to the chicken genome using the pangenome [11] as a reference. A total of 89.7% of the read pairs were uniquely mapped, and 6.43% were ambiguously mapped due to genomic repeats. The remaining sequences were classified into chimeric (0.87%), either due to structural variants or PCR template switching artefacts; unmapped, due to low base call quality (0.59%); and novel sequences, partially mapped to the pangenome (1.64%) or fully unmapped (0.76%). This preliminary analysis showed a high sequencing quality and the existence of a small number of novel sequences missing from the reference genome. Analysis of the unmapped reads showed the presence of a small amount of contamination. Among the unmapped reads, there is rat and *Neogale vison* DNA, which we explain by rare errors in the read demultiplexing (reads related to the samples sequenced in the same run). In addition, the samples contained avian viruses, *Galliform chaphamaparvovirus*, *Avian endogenous retrovirus EAV-HP*, and *Rous Sarcoma virus*, that appear to be located in the tissues under study or directly in the chicken genome. Furthermore, we identified bacterial 16S rRNA of unknown origin. The remaining unmapped reads can be successfully mapped to the genomes of other bird species, and apparently belong to the genomic regions of chickens missed during the sequencing of other chicken breeds (due to the high GC and/or G4s), or to the genomic regions specific to the RSW breed.

Mapping to the pangenome [11] was performed with bowtie2 v2.2.3 [32] in paired-end mode, allowing up to 1000 bp inserted between the read pairs. The mapping statistics were collected using an in-house program (https://github.com/yevshin/bioutils accessed on 1 September 2024). Analysis of the unmapped reads was conducted by mapping them with Blast [33] to the nt database.

The de novo contigs were assembled using the MASURCA v4.1.0 genome assembler [34] with the parameters listed in [Supplementary Table S7]. The contig to chromosome assignment and ordering was conducted using the reference-guided scaffolder RAGTAG v2.1.0 [35,36], with the “-r -g 1” parameters. To clear the non-bird sequences from the unmapped contigs, we aligned them to the nt database with blastn v2.9.0+ [33].

Due to the relatively high coverage of the mitochondrial genome compared to the nuclear chromosomes, MASURCA was not able to recover the complete mitochondrial chromosomes when assembling from all input reads. The mitochondrial genome was assembled separately. To save computer time, 10 million subsets of raw reads were selected and these reads were aligned to the Huxu mitochondrial genome, allowing sequence boundary crossing with the minimap2 v2.26-r1175 aligner [37] in “-ax sr” mode. The mapped reads were collected and used for the de novo assembly with MASURCA. Artificial overhangs, which appeared due to the circular nature of the mitochondrial genome, were manually removed.

To access the completeness of the assembly based on the analysis of orthologous genes, we used BUSCO v5.7.0 [38] in the genomic mode (“-m genome”) with the augustus gene predictor (“-augustus”) and the “aves_odb10.2024-01-08” ortholog gene dataset.

The gene annotation of the GGRsw1 genome was performed by mapping the existing annotation of the GRCg7b assembly using the Liftoff v1.6.3 tool [39].

To identify the heterozygous sites of the sequenced chickens, raw reads were mapped to the GGRsw1 assembly using bowtie2; the alignments with mapq ≥ 10 were selected and used for variant calling. The variant calling was conducted by the standard bcftools v1.8 pipeline [40]. The variant calls with QUAL < 200 were filtered out and the remaining variants were converted into the BCF file format.

The pairwise alignment of the genomic sequences of GGRsw1 with the 33 chicken genomes of the other breeds was performed using winnowmap v2.03 [41,42].

For the phylogenetic analysis, SNPs were called based on the pairwise whole-genome alignments between GGRsw1 and each of the 33 chicken genomes. We restricted the analysis to the genomic regions present in the all of studied genomes. In total, we identified 11.7 million such SNPs. The phylogenetic tree was built using RaxML [43] v8.2.12 under the GTRGAMMA substitution model. Bootstrap analysis with RaxML showed a high confidence of all branches (Internode Certainty > 80). The resulting tree was rooted at the Red Junglefowl and plotted using the APE v5.8 [44] R package.

To identify the RSW-specific mutations, genomic variants distinguishing the breeds were called based on the whole-genome alignments. Due to the incompleteness of some genomic assemblies, we studied the regions represented in at least 30 of the 33 assemblies. If a particular allele was found only in the RSW genome and not in any other genome, it was classified as RSW-specific.

### 4.1. Test for Enrichment of RSW-Specific Mutations

By pairwise-comparing the RSW genome with many genomes of other chicken breeds, we find many mutations that distinguish the corresponding breeds. Some of these mutations are unique to the RSW; that is, they are not found in the other breeds, and are of particular interest. An increased frequency of unique mutations in a gene, group of genes (genes from the same GO term), or genomic region may indicate the genetic processes occurring in the RSW. The accumulation of mutations occurs unevenly throughout the genome, so when searching for genes (groups of genes or genomic regions) with an increased frequency of unique mutations, the appropriate statistical methods must be used. Consider the set S of genomic positions on the RSW genome that have a nucleotide difference compared to one of the other breeds. This set S may be a set of mutations of a particular gene, a group of genes with similar functions, or a particular region of a genome. Some of these positions are unique to the RSW in the sense that such a nucleotide is found only in the RSW breed. Let us denote the set of such unique mutations by U. Then, the proportion of unique mutations will be *p* =|U|/|S|. Let us set S’ as a subset of S, with a corresponding set U’; for example, S’ can be a set of the mutations of one gene, all mutations of the genes belonging to a given GO category, or the mutations of a certain genomic region. As a null hypothesis, we use the statement that the proportion of unique mutations is the same in S and S’. In practice, S is sufficiently large, so |U’| has a binomial distribution,

*p*-value = P(|U’| >= *k*) = pbinom(*p*, *n*, *k*), (1)

where the success probability *p* = |U|/|S|, the number of trials *n* = |S’|, and the number of successes *k*. We consider the excess of the proportion of unique mutations significant if the *p*-value < 0.001, and additionally impose a filter on the effect size.

fold change = (|U’|/|S’|)/(|U|/|S|) >= 2.(2)

The applications of this method for various tasks include the following:

- Searching for genes with an increased frequency of RSW-specific missense mutations. S is the set of all missense mutations of all genes, and S’ is the set of missense mutations of a given gene;
- Searching for genes with an increased frequency of RSW-specific mutations near a gene (gene bounds ±10 kbp). S is the set of all mutations located near any gene, and S’ is the set of mutations near the given gene;
- Searching for GO categories with an increased frequency of missense mutations. S is the set of all missense mutations of all genes from all GO categories, and S’ is the set of missense mutations of all genes of a given GO category;
- Searching for GO categories with a high rate of RSW-specific mutations in a ±10 kbp window from the gene bounds. S is the set of all mutations located within the boundaries of genes ±10 kbp of all GO categories; S’ is the set of all mutations located within the boundaries of genes ±10 kbp of a given GO category.

## Figures and Tables

**Table 1:** Genes with high rate of nonsynonymous mutations in Russian Snow White.

Protein	Mutations	Gene
actin filament-associated protein 1	3	*AFAP1*
homeobox protein BarH-like 1	2	*BARX1*
protein CDV3 homolog	2	*CDV3*
neural cell adhesion molecule L1-like protein	4	*CHL1*
probable ATP-dependent RNA helicase DDX31	4	*DDX31*
DNA excision repair protein ERCC-6-like 2	2	*ERCC6L2*
protein FAM168A	2	*FAM168A*
COP9 signalosome complex subunit 1	2	*GPS1*
grainyhead-like protein 2 homolog	2	*GRHL2*
hemicentin-1	3	*HMCN1*
Phosphatidylinositol 3,4,5-trisphosphate 5-phosphatase 1	3	*INPP5D*
T-cell receptor alpha variable 20-like	5	*LOC107049836*
uncharacterized protein LOC107051951	3	*LOC107051951*
uncharacterized protein LOC121107430	4	*LOC121107430*
mitochondrial antiviral signaling protein	3	*MAVS*
mediator of RNA polymerase II transcription subunit 1	3	*MED1*
mitochondrial genome maintenance exonuclease 1	2	*MGME1*
serine/threonine-protein kinase *NEK10*	4	*NEK10*
neurogenic locus notch homolog protein 2 precursor	3	*NOTCH2*
P2X purinoceptor 7	3	*P2RX7*
3-phosphoinositide-dependent protein kinase 1	2	*PDPK1*
PEX5-related protein	2	*PEX5L*
E3 SUMO-protein ligase PIAS2	2	*PIAS2*
plakophilin-4	6	*PKP4*
PLAC8 like 2	2	*PLACL2*
DNA polymerase lambda	3	*POLL*
mothers against decapentaplegic homolog 9	2	*SMAD9*
synaptic vesicle glycoprotein 2C	2	*SV2C*
tyrosine 3-monooxygenase	3	*THL*

**Table 2:** Gene ontology categories related to TGF-beta signaling with increased frequency of mutations specific to Russian Snow White breed.

*p*-Value	Fold Change	GO Category
2 × 10^−4^	5.9	regulation of transforming growth factor beta receptor signaling pathway
2.2 × 10^−4^	5.9	regulation of cellular response to transforming growth factor beta stimulus
9.2 × 10^−5^	4.5	transforming growth factor beta receptor signaling pathway
5.1 × 10^−5^	4.4	cellular response to transforming growth factor beta stimulus
5.5 × 10^−5^	4.4	response to transforming growth factor beta
4.1 × 10^−5^	3.3	transforming growth factor beta receptor superfamily signaling pathway
4.2 × 10^−4^	4.1	regulation of transmembrane receptor protein serine/threonine kinase signaling pathway
2.4 × 10^−4^	3.3	transmembrane receptor protein serine/threonine kinase signaling pathway

**Table 3:** The genes of the TGF-beta pathway specifically mutated in the Russian Snow White breed.

CDS Length	Number of RSW-Specific Missense Mutations	Description	Gene
2526	2	transforming growth factor beta receptor type 3 precursor	*TGFBR3*
3801	1	histone-lysine N-methyltransferase PRDM16	*PRDM16*
3645	2	zinc finger E-box-binding homeobox 2	*ZEB2*
3279	1	E3 ubiquitin-protein ligase TRIM33	*TRIM33*
1959	1	endoplasmic reticulum chaperone BiP precursor	*HSPA5*
1680	1	paxillin	*PXN*
2163	2	fermitin family homolog 2	*FERMT2*
2454	1	transcription factor SOX6	*SOX6*
978	1	protein TOB1	*TOB1*
1158	1	protein pelota homolog	*PELO*
7089	1	spectrin beta chain non-erythrocytic 1	*SPTBN1*

**Table 4:** The gene ontology categories related to the immune system with an increased frequency of mutations specific to the Russian Snow White breed near the genes (±10 kbp from the gene boundaries).

*p*-Value	Fold Change	GO Category
<1 × 10^−6^	5.36	MHC class II protein complex
<1 × 10^−6^	5.36	MHC protein complex
<1 × 10^−6^	5.03	antigen binding
<1 × 10^−6^	4.8	MHC protein complex binding
<1 × 10^−6^	4.61	peptide antigen assembly with MHC protein complex
<1 × 10^−6^	4.61	MHC protein complex assembly
<1 × 10^−6^	3.89	antigen processing and presentation of peptide or polysaccharide antigen via MHC class II
<1 × 10^−6^	3.5	antigen processing and presentation of exogenous antigen
<1 × 10^−6^	3.13	antigen processing and presentation of peptide antigen
<1 × 10^−6^	2.34	immunoglobulin mediated immune response
<1 × 10^−6^	2.29	antigen processing and presentation
<1 × 10^−6^	2.18	B-cell mediated immunity
<1 × 10^−6^	2.12	regulation of B-cell activation

**Table 5:** The genes related to the immune system that have an RSW-specific mutation in the gene region (±10 kb from the gene boundaries).

Number of RSW-Specific Mutations ±10 kb of Gene	Description	Gene
16	MHC class II M beta chain 1	*DMB1*
8	Major histocompatibility complex class II beta chain BLB1	*BLB1*
2	class II histocompatibility antigen, B-L beta chain	*MHCY2B2*
16	major histocompatibility complex, class II, DM beta 2	*DMB2*
17	B locus M alpha chain 1 precursor	*DMA*
19	beta-2-microglobulin precursor	*B2M*
15	uncharacterized protein LOC101747454 precursor	*BLB2*
5	antigen peptide transporter 1	*TAP1*
14	T-cell surface glycoprotein CD1B precursor	*CD1B*
11	tapasin isoform 1 precursor	*TAPBP*
2	protein disulfide-isomerase A3 precursor	*PDIA3*
1	ADP-ribosylation factor-like protein 8B	*ARL8B*
6	TNF receptor-associated factor 6	*TRAF6*
8	major facilitator superfamily domain-containing protein 6	*MFSD6*
11	telomere-associated protein RIF1	*RIF1*
21	complement component C6	*C6*
5	interleukin-2 receptor subunit beta precursor	*IL2RB*
31	protein kinase C delta type	*PRKCD*
12	complement component C8 alpha chain	*C8A*
1	tyrosine-protein phosphatase nonreceptor type 6	*PTPN6*
7	Sushi, von Willebrand factor type A, EGF, and pentraxin domain-containing protein 1	*CR1*
4	C-C chemokine receptor type 6	*CCR6*
77	B-cell lymphoma 6 protein homolog	*BCL6*
12	transferrin receptor protein 1	*TFRC*
8	complement component C7 precursor	*C7*
2	interleukin-21 receptor	*IL21R*
13	60 kDa heat shock protein, mitochondrial precursor	*HSPD1*
1	DNA mismatch repair protein *MLH1*	*MLH1*
5	complement C2	*C2*
8	CD81 antigen	*CD81*
1	uracil-DNA glycosylase	*UNG*
1	HLA class II histocompatibility antigen gamma chain	*CD74*
1	C-C chemokine receptor type 7	*CCR7*
5	nucleotide-binding oligomerization domain-containing protein 1	*NOD1*
22	ras-related protein *RAB3C*	*RAB3C*
13	autophagy protein 5	*ATG5*
10	WD repeat- and FYVE domain-containing protein 4	*WDFY4*
14	T-cell surface glycoprotein *CD1B* precursor	*CD1B*
2	protein disulfide-isomerase A3 precursor	*PDIA3*
11	telomere-associated protein RIF1	*RIF1*
6	netrin-1 precursor	*NTN1*
12	complement component C8 alpha chain	*C8A*
4	C-C chemokine receptor type 6	*CCR6*
2	interleukin-6 precursor	*IL6*
2	interleukin-21 precursor	*IL21*
4	tyrosine-protein kinase SYK	*SYK*
2	DNA mismatch repair protein *MSH6*	*MSH6*
1	tumor necrosis factor receptor superfamily member 13B	*TNFRSF13B*
1	toll-like receptor 4 precursor	*TLR4*
70	SAM domain-containing protein *SAMSN1*	*SAMSN1*
4	nuclear factor of activated T cells, cytoplasmic 2	*NFATC2*
14	mRNA decay activator protein ZFP36L1	*ZFP36L1*
1	cytotoxic T-lymphocyte protein 4 precursor	*CTLA4*
8	apoptosis regulator *BCL2*	*BCL2*
26	serine/threonine-protein phosphatase 2A regulatory subunit B″ subunit gamma	*PPP2R3C*

**Table 6:** Missense mutations near chr2:119–124 Mb locus that are unique to Russian Snow White. Genes within considered locus are in bold.

Codon and Amino Acid Changed	Type	Gene	GGRSw1 Coordinates
tgc > cgc c > r at 1494	NONSYNONYMOUS_SNV	*STAU2*	chr2:118293681:A > G
	SPLICE_SITE_VARIANT	*EYA1*	chr2:117404520:T > C
aca > aaa t > k at 6745	NONSYNONYMOUS_SNV	***ZFHX4***	chr2:119867016:C > A
cgt > ctt r > l at 643	NONSYNONYMOUS_SNV	***RALYL***	chr2:122881444:G > T

**Table 7:** Genomic regions previously identified as under selection pressure in Russian White with increased density of RSW-specific mutations.

Density Fold Change	Number of RSW-Specific Variants	Study	Genomic Region, GGRsw1 Coordinates
1.45	44	Abdelmanova 2021 [7]	chr2: 79330796-79662493
4.1	11	Fedorova 2022 [3]	chr2: 27306204-27335181
4.72	12	Fedorova 2022 [3]	chr1: 45987922-46015642
1.68	62	Romanov 2023 [4]	chr4: 39155998-39558429
1.69	71	Romanov 2023 [4]	chr4: 70521103-70978718
1.83	56	Romanov 2023 [4]	chr4: 85444734-85777940
1.86	105	Romanov 2023 [4]	chr3: 42284613-42897825
1.91	189	Romanov 2023 [4]	chr9: 22641704-23718280
2.04	75	Romanov 2023 [4]	chr4: 12025118-12426293
2.18	81	Romanov 2023 [4]	chr13: 13487650-13891703
2.23	82	Romanov 2023 [4]	chr9: 17772796-18172350
2.65	98	Romanov 2023 [4]	chr5: 43870263-44272742

## Data Availability

Genome assembly and annotation files are available from https://chicken.biouml.org/downloads/ChickenResearch2023/RussianSnowWhite/ (accessed on 1 August 2024).

## Supplementary Materials

The following supporting information can be downloaded at https://www.mdpi.com/article/10.3390/ijms252011066/s1.
